# Novaluron Has Detrimental Effects on Sperm Functions

**DOI:** 10.3390/ijerph19010061

**Published:** 2021-12-22

**Authors:** Ju-Mi Hwang, Jeong-Won Bae, Eun-Ju Jung, Woo-Jin Lee, Woo-Sung Kwon

**Affiliations:** 1 Department of Animal Science and Biotechnology, Kyungpook National University, Sangju 37224, Korea; ghkdwnal100@gmail.com (J.-M.H.); jwbae1822@gmail.com (J.-W.B.); wj9059lee@naver.com (W.-J.L.); 2 Department of Animal Biotechnology, Kyungpook National University, Sangju 37224, Korea; red0787@naver.com

**Keywords:** novaluron, sperm function, capacitation, spermatozoa

## Abstract

Although novaluron is an insect growth regulator with a low mammalian acute toxicity and a low risk to the environment and nontarget organisms, toxic effects of novaluron have been reported. However, no studies have yet evaluated the effect of novaluron on reproduction. Therefore, we examined the effects of novaluron on sperm functions. The spermatozoa of ICR mice were incubated with various concentrations of novaluron to induce capacitation. Then, sperm motion parameters and capacitation status were evaluated using CASA program and H33258/chlortetracycline staining. In addition, PKA activity and tyrosine phosphorylation were evaluated by Western blotting. After exposure, various sperm motion parameters were significantly decreased in a dose-dependent manner. The acrosome reaction was also significantly decreased in the high concentration groups. Sperm viability was significantly reduced at the highest concentration. In addition, PKA activity and tyrosine phosphorylation were also significantly altered. Thus, novaluron affects sperm viability, sperm motility, and motion kinematics during capacitation. Furthermore, it may promote the reduction in acrosome reactions. The physiological suppression of sperm function may depend on abnormal tyrosine phosphorylation via the alteration of PKA activity. Therefore, we suggest that it is necessary to consider reproductive toxicity when using novaluron as a pesticide.

## 1. Introduction

Novaluron belongs to a new class of insecticide known as benzoylphenylurea that is currently registered for the control of thrips, whiteflies, leaf miners, and other foliar-feeding insect pests of ornamental plants grown in greenhouses [1]. Novaluron is anticipated to decrease the dependence on organophosphates such as diazinon, pyrethroids, and carbamate insecticides [1]. The mode of action of novaluron is not well documented, but the general mechanisms and effects are similar to those of benzoylphenylureas, which inhibit the biochemical processes, resulting in the formation of chitin synthetase, causing an abnormal deposition of the endocuticle [2]. This insecticide primarily affects the larval stages, causing death by abnormal endocuticular deposition and disturbing molting, and hence, benzoylphenylurea compounds are known as chitin synthesis inhibitors (CSIs). It has low mammalian acute toxicity and reduced risks to the environment and nontarget organisms and thus considered as acceptable for inclusion in integrated pest management programs [3,4]. For this reason, CSIs, such as novaluron, have become alternatives to conventional insecticides and have been widely used in agriculture. In 2011, the total CSI (benzoylphenylurea) global market share of insecticides was 3.6% [5].

Although novaluron is a known low-risk chemical, several researchers have reported that it has a hazardous effect on mammals [6,7,8]. Studies have reported that benzoylphenylurea family-like novaluron affects fetal body weight loss, color change of urine and skin, liver function, and skeletal abnormality after maternal exposure [6,7]. It has also been demonstrated that benzoylphenylurea family-like novaluron exhibited hazardous effects on male reproduction, such as decreases in testis weight, daily sperm production, and number of sperms in the epididymis [8]. However, no study has yet evaluated the reproductive toxicity of novaluron directly.

For successful fertilization, ejaculated sperm cells must undergo a special process within the female reproductive tract to acquire the full capacity to fertility, which has been termed “capacitation” [9,10]. In this process, sperm motility patterns change, and the sperm undergoes alterations in various molecular functions, such as protein kinase A activation and tyrosine phosphorylation [11,12,13,14,15,16]. Finally, the capacitated sperm cells undergo an acrosomal change, i.e., the acrosome reaction, after which, the acrosome-reacted sperm cell can penetrate the oocyte [11,12,13,14,15,16]. Therefore, the present study was conducted to evaluate the male reproductive toxicity of novaluron by exploring the sperm functions after inducing capacitation.

## 2. Materials and Methods

### 2.1. Chemicals and Media

Modified Tyrode’s medium was used as the basic medium (BM) (97.84 mM NaCl, 1.42 mM KCl, 0.47 mM MgCl_2_·6H_2_O, 0.36 mM NaH_2_PO_4_·H_2_O, 5.56 mM _D_-glucose, 25 mM NaHCO_3_, 1.78 mM CaCl_2_·2H_2_O, 24.9 mM Na-lactate, 0.47 mM Na-pyruvate, 50 µg/mL gentamicin, and 0.005 mM phenol red). To induce sperm capacitation, 0.4% bovine serum albumin was added to the BM. All chemicals were purchased from Sigma-Aldrich (St Louis, MO, USA).

### 2.2. Preparation and Treatment of Spermatozoa

All animal procedures were performed according to the guidelines for the ethical treatment of animals and were approved by the Institutional Animal Care and Use Committee of Kyungpook National University (KNU 2017-141). All animals were maintained at a temperature of 24 °C ± 2 °C under a 12 h light/dark cycle with appropriate humidity. The animals were provided food (Cargil Agripurina, Seongnam, Korea) and water ad libitum. Male ICR mice (Nara Biotech, Seoul, Korea) were sacrificed at 8–12 weeks of age by cervical dislocation. Spermatozoa were released from the cauda epididymis into BM containing 0.4% BSA. Then, the spermatozoa were incubated with various concentrations of novarulon at 37 °C and 5% CO_2_ for 90 min to induce capacitation as described previously [11,12,13,14,15]. The concentrations of novarulon were considered by referring to previous studies [17,18].

### 2.3. Sperm Motility and Motion Kinematics

The computer-assisted sperm analysis program (CASA) (FSA2016, Medical Supply, Seoul, Korea) equipped with a CMOS image sensor and a 2048 × 1536 (300 M pixels), 60-frame camera (Medical Supply, Seoul, Korea), and an OLYMPUS BX43 phase-contrast microscope (Olympus, Tokyo, Japan) with a 10× objective phase-contrast mode, was used to evaluate sperm motility and motion kinematics. A 10 µL smear of sample was evaluated in a preheated (37 °C) Makler counting chamber (Sefi-Medical Instruments, Haifa, Israel). Total sperm motility (%), rapid sperm motility (%), medium sperm motility (%), slow sperm motility (%), progressive sperm motility (%), curvilinear velocity (VCL, µm/s), straight-line velocity (VSL, µm/s), average path velocity (VAP, µm/s), beat cross frequency (BCF, Hz), and mean amplitude of head lateral displacement (ALH, μm) were measured.

### 2.4. Evaluation of Cell Viability

To monitor cellular functions, a cell cytotoxicity assay was performed using the Cell Cytotoxicity Assay Kit (Abcam, Cambridge, UK). Before experiments, the assay solution was thawed and preheated to 37 °C. Sample wells were incubated at 37 °C and 5% CO_2_ for 2 h protected from light. After gently shaking the plates, the absorbance was monitored at OD 570 and 650 nm (Gemini Em; Molecular Devices Corporation, Sunnyvale, CA, USA). Cell viability was measured using the ratio of OD 570 nm to OD 650 nm (SoftMax Pro 7; Molecular Devices Corporation, Sunnyvale, CA, USA).

### 2.5. Assessment of Sperm Capacitation Status

The dual staining method (combined Hoechst 33258/chlortetracycline fluorescence assessment) was used to evaluate capacitation status [11,12,13,14,15]. Samples were incubated for 90 min and then centrifuged at 100× *g* for 2.5 min. Most of the supernatant was removed, and samples were kept in 135 µL, after which, 15 μL of H33258 solution (10 μg H33258/mL DPBS) was added to the remaining 135 µL of samples and incubated for 2 min at RT. Then, 250 μL of 2% (*w*/*v*) polyvinylpyrrolidone in DPBS was added, and the sample was centrifuged at 100× *g* for 2.5 min. The supernatant was completely removed, and the samples were resuspended in 100 μL of chlortetracycline fluorescence (CTC) solution (750 mM CTC in 5 μL buffer: 20 mM Tris, 130 mM NaCl, and 5 mM cysteine, pH 7.4) and 100 μL of DPBS. After incubating the samples in the refrigerator without light for 20 min, 10 μL of each sample was smeared onto slides. At least 400 spermatozoa were evaluated per slide for each sample. An OLYMPUS BX43 microscope under epifluorescence illumination using ultraviolet BP 340–380/LP 425 and BP 450–490/LP 515 excitation/emission filters were used for H33258 and CTC (Olympus, Tokyo, Japan). Finally, the samples were evaluated and divided into four groups according to the capacitation status, as follows: live acrosome-reacted sperm (AR pattern), live capacitated sperm (B pattern), live non-capacitated sperm (F pattern), and dead sperm (D pattern).

### 2.6. Western Blot Analysis of Phospho-PKA Substrates and Tyrosine Phosphorylation

Phospho-PKA substrates and tyrosine phosphorylation were examined by Western blot analysis. Each sample group was washed twice with DPBS and centrifuged at 10,000× *g* for 5 min. Sperms were stored in modified Laemmli sample buffer (315 mM Tris, 10% glycerol, 10% SDS, 5% 2-mercaptoethanol, 5% bromophenol blue, HPLC) at RT for 10 min and then centrifuged at 10,000× *g* for 5 min. The resulting supernatants were boiled at 95 °C for 3 min. The samples were then separated using a 12% SDS-PAGE gel (MiniPROTEIN Tetra Cell, Bio-Rad, Hercules, CA, USA), and then the separated proteins were transferred onto PVDF membranes (Bio-Rad, Hercules, CA, USA). The membranes were incubated at RT for 2 h in a 3% ECL blocking agent (GE Healthcare, Chicago, IL, USA). Next, the membranes were washed two times with PBS-T and incubated with the primary antibody overnight at 4 °C. Primary antibodies were diluted with the 3% ECL blocking agent (phospho-PKA substrate antibody (Cell Signaling Technology, Danvers, MA, USA), 1:1000; anti-phosphotyrosine antibody [PY20] (HRP) (Abcam, Cambridge, UK), 1:5000; anti-alpha tubulin antibody (Abcam, Cambridge, UK), 1:5000). After treatment, the membranes were washed four times with PBS-T. Goat anti-rabbit IgG H&L (HRP) was diluted at 1:2000 with the 3% ECL blocking agent and used as a secondary antibody for phospho-PKA substrates. Goat anti-rabbit IgG H&L (HRP), diluted with the 3% ECL blocking agent at 1:2000, was used as a secondary antibody for α-tubulin. Protein expression levels were detected by Image Quant LAS 500 (GE Healthcare, Chicago, IL, USA), using the enhanced chemiluminescence (ECL) method. Then, the Image Studio Lite (Version 5.0, LI-COR Corporate, Lincoln, NE, USA) system was used for expression analysis. The density ratios of each treatment band were normalized to α-tubulin.

### 2.7. Statistical Analysis

One-way ANOVA using the SPSS software (Version 25.0, IBM, Armonk, NY, USA) was applied for the statistical analysis of all data. Tukey’s multiple comparison test was used to compare the five groups with the control. Each experiment was performed at least four times. Numerical data are expressed as mean ± SEM. The level of significance was set at *p* < 0.05.

## 3. Results

### 3.1. Effects of Novaluron on Sperm Total Motility and Motion Kinematics

The CASA system was used to evaluate sperm motility and motion kinematics. Total motility, rapid motility, progressive motility, VCL, and ALH were significantly decreased at high concentrations of novaluron (1, 10, and 100 µM) (*p* < 0.05; Table 1). VAP and BCF were significantly decreased at 10 and 100 µM concentrations of novaluron (*p* < 0.05; Table 1). VSL was also significantly decreased at the highest novaluron concentration (100 µM) (*p* < 0.05; Table 1). Medium motility and slow motility showed no significant alterations (Table 1).

### 3.2. Cell Viability

**Figure 1:**
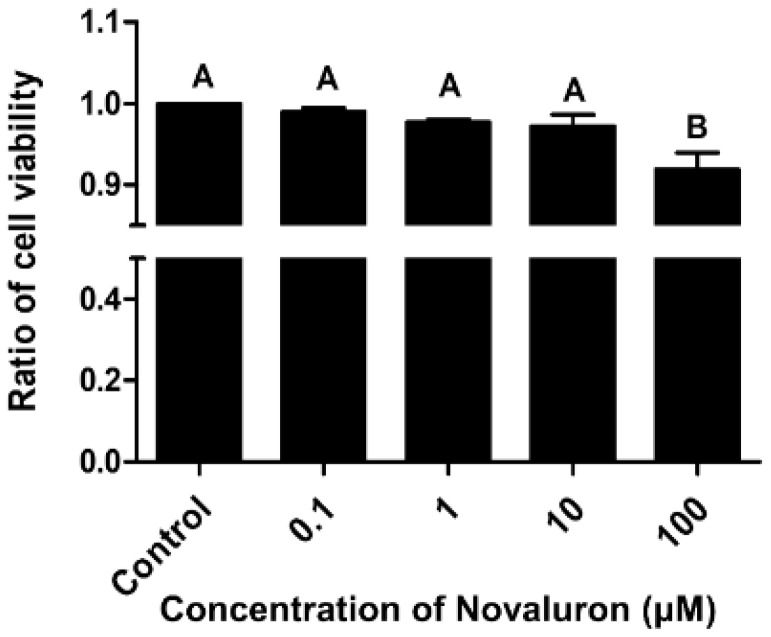
Effects of novaluron on cell viability. Cell viability after exposure to different concentrations of novaluron. Data represent mean ± SEM, *n* = 4. Data were evaluated by one-way ANOVA and represent mean ± SEM, *n* = 4. Values with different superscripts (^A,B^) indicate significant differences between the control and treatment groups (*p* < 0.05).

Cell viability was decreased in a dose-dependent manner. However, significant differences were observed at the highest concentration of novaluron (100 µM) (*p* < 0.05; Figure 1).

### 3.3. Assessment of Sperm Capacitation Status

**Figure 2:**
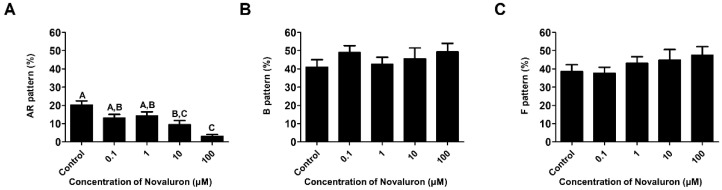
Effects of novaluron on capacitation status. (**A**) Patterns of live and acrosome-reacted sperms (AR pattern). (**B**) Patterns of live and capacitated sperms (B pattern). (**C**) Patterns of live and non-capacitated sperms (F pattern). Data were quantified by one-way ANOVA and represent mean ± SEM, *n* = 4. Values with different superscript letters (^A,B,C^) indicate significant differences between the control and treatment groups (*p* < 0.05).

H33258/CTC dual staining was performed to investigate the capacitation status. The number of acrosome reacted spermatozoa was significantly decreased when treated with high concentrations of novaluron (10 and 100 µM) (*p* < 0.05; Figure 2A). However, there were no significant alterations in the number of capacitated and non-capacitated spermatozoa (Figure 2B,C).

### 3.4. PKA Activity and Tyrosine Phosphorylation

**Figure 3:**
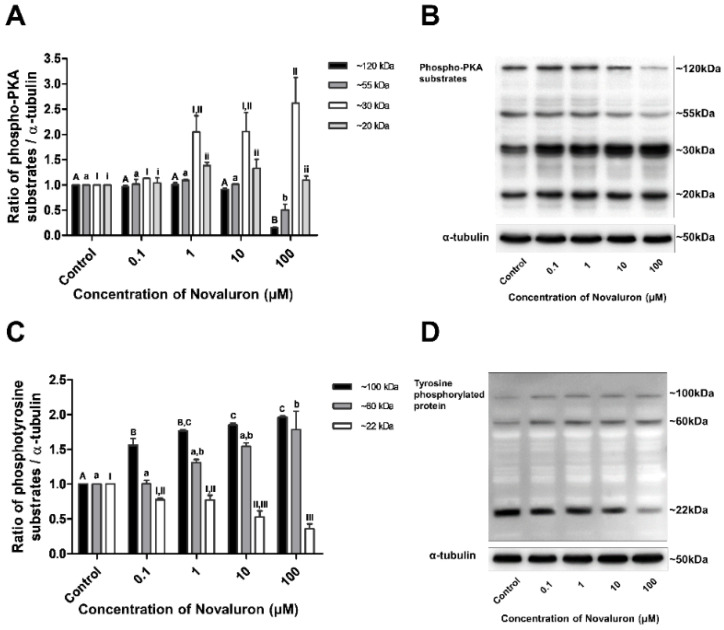
Effect of novaluron on PKA activity and protein tyrosine phosphorylation. (**A**) Phospho-PKA substrate density was measured at approximately 120, 55, 30, and 20 kDa. (**B**) Phospho-PKA substrates were probed. Lane 1: control; Lane 2: 0.1 µM novaluron; Lane 3: 1 µM novaluron; Lane 3: 10 µM novaluron; Lane 5: 100 µM novaluron. (**C**) The density of phosphotyrosine protein substrates was measured at approximately 100, 60, and 22 kDa. (**D**) Phosphotyrosine protein substrates were probed. Lane 1: control; Lane 2: 0.1 µM novaluron; Lane 3: 1 µM novaluron; Lane 4: 10 µM novaluron; Lane 5: 100 µM novaluron. Data were evaluated by one-way ANOVA and represent mean ± SEM, *n* = 4. Values with different superscripts (^A,B,C,a,b,I,II,III,i,ii^) indicate significant differences between the control and treatment groups (*p* < 0.05).

The levels of phospho-PKA substrates and tyrosine phosphorylation were measured by Western blotting. Phospho-PKA substrates were found at approximately 20, 30, 55, and 120 kDa, and phosphotyrosine substrates were detected at approximately 22, 60, and 100 kDa. The levels of approximately 20 and 30 kDa phospho-PKA substrates were significantly increased, whereas those of approximately 55 and 120 kDa substrates were significantly decreased in a dose-dependent manner (*p* < 0.05; Figure 3A,B). The levels of approximately 22 kDa phosphotyrosine substrates were significantly decreased, whereas those of approximately 60 and 100 kDa substrates were significantly increased in a dose-dependent manner (*p* < 0.05; Figure 3B,C).

## 4. Discussion

Selectively effective biorational insecticides are considered to have less risk to beneficial organisms compared with broad-spectrum pesticides [19]. The general mechanism of benzoylphenylureas is to inhibit the biochemical processes, leading to the formation of chitin synthetase, causing an abnormal deposition of the endocuticle [2]. For this reason, chitin synthesis inhibitors (CSIs), such as novaluron, have become alternatives to conventional insecticides and have been widely used in agriculture because the CSIs do not kill the pests based on directly toxic effects. In 2011, CSIs’s total global market share of insecticide was 3.6% and the market is expected to expand every year [5]. Novaluron, among various CSIs, is an effective biorational insecticide and used worldwide, and the Novaluron is anticipated to decrease the reliance on organophosphates, such as diazinon, pyrethroids, and carbamate insecticide, which are broad-spectrum pesticides [1]. Although novaluron has become an alternative to conventional insecticides and is widely used in agriculture, the issue of the toxic effect of novaluron has emerged continuously. Studies have reported that exposure to benzoylphenylurea family-like novaluron induces various toxic effects [6,7,8]. Although various risks of novaluron have been reported, its effects on male reproductive functions are not clear. Therefore, to determine the effects of novaluron on male fertility, we investigated various sperm functions in mouse spermatozoa after treatment with novaluron.

Sperm motion patterns change to vigorous patterns during capacitation [20,21]. Therefore, the changes in the sperm motion parameters are an important factor for a successful pregnancy and are thus widely used to predict spermatozoa capability for fertilization [11,12]. In the present study, the CASA system was used to evaluate various sperm motility patterns and motion kinematic parameters. The sperm motility patterns were evaluated in terms of total, rapid, medium, slow, and progressive motility, separately. Furthermore, the motion kinematic parameters VCL, VSL, VAP, BCF, and ALH were evaluated. Results showed that sperm motility and motion kinematic parameters were suppressed by novaluron treatment. Hence, exposure to novaluron may induce adverse effects on male fertility by decreasing sperm motility and motion kinematics during capacitation. Cell viability was also significantly decreased at the highest concentration of novaluron. This may be one of the reasons for the decreases in most sperm motility and motion kinematic parameters at the highest concentration of novaluron.

For successful fertilization, the ejaculated spermatozoa must undergo capacitation during their passage through the female genital tract [9,21]. Capacitation is initiated by various factors, such as BSA, heparin, glucose, and glycosaminoglycan. Various enzymes are activated by the calcium cation influx. Finally, tyrosine phosphorylation occurs [22,23]. Only the acrosome reacted sperm cells can penetrate the zona pellucida after capacitation [9,10,24,25]. In the present study, as PKA activity is one of the important enzymatic activities that induces tyrosine phosphorylation, tyrosine phosphorylation was analyzed to determine the effects of novaluron on the molecular changes occurring during capacitation. Interestingly, the levels of ~20 and 30 kDa PKA substrates were increased and those of ~55 and 120 kDa PKA substrates were decreased in a dose-dependent manner. Furthermore, the levels of ~22 kDa phosphotyrosine proteins were decreased and those of ~60 and 100 kDa tyrosine phosphorylated proteins were increased in a dose-dependent manner. Tyrosine phosphorylation is generally induced by PKA activation during capacitation in spermatozoa [11,22,23]. However, our results showed that novaluron reduces the levels of ~55 and 120 kDa phosphor substrates. This may be induced by increased levels of other PKA substrates (~20 and 30 kDa). Moreover, abnormal alterations were observed in tyrosine phosphorylation. Our results suggest that unusual changes in PKA activity induce abnormal tyrosine phosphorylation. However, to more accurately examine the effect of novaluron on PKA activity and tyrosine phosphorylation, further studies are required to analyze the molecular mechanism of specific proteins involved in tyrosine phosphorylation during capacitation. Moreover, the detrimental effect of novaluron on the acrosome reaction was observed in the present study. This suggests that the acrosome reaction is suppressed by the abnormal induced capacitation due to irregular PKA activation and tyrosine phosphorylation. Therefore, our results suggest that novaluron exerts a harmful effect on PKA sensitivity and tyrosine phosphorylation, which eventually induces abnormal capacitation, thereby adversely affecting the acrosome reaction.

## 5. Conclusions

Novaluron is well known as one of the suitable pesticides worldwide because of its low mammalian acute toxicity and reduced risks to the environment and nontarget organisms. However, our results demonstrated that novaluron induces detrimental effects on sperm functions during capacitation, and, to our knowledge, this is the first report concerning the male reproductive toxicity of novaluron. In particular, abnormal PKA activity and tyrosine phosphorylation were observed, resulting in the suppression of sperm motility, motion kinematics, cell viability, and the acrosome reaction. Therefore, we suggest that novaluron should be used considering its reproductive toxicity, and in particular, it is essential to warn people who have occupations related to novaluron use, regarding its reproductive toxicity. In addition, further studies are needed to evaluate the exact concentration for the safe use of novaluron.

## Figures and Tables

**Table 1:** Effects of novaluron on sperm motility and motion kinematics.

	Concentration of Novaluron (µM)				
	Control	0.1	1	10	100
MOT (%)	65.28 ± 1.89 ^a^	55.84 ± 1.70 ^a,b^	51.93 ± 2.61 ^b^	52.24 ± 1.74 ^b^	38.23 ± 4.49 ^c^
Rapid (%)	48.73 ± 2.13 ^a^	40.30 ± 1.60 ^a,b^	36.91 ± 1.97 ^b^	37.27 ± 2.99 ^b^	25.11 ± 3.61 ^c^
Medium (%)	16.36 ± 1.04	15.19 ± 0.53	14.64 ± 1.27	14.45 ± 1.68	12.90 ± 1.32
Slow (%)	0.17 ± 0.11	0.31 ± 0.10	0.38 ± 0.14	0.52 ± 0.24	0.14 ± 0.06
Progressive (%)	65.10 ± 1.84 ^a^	55.49 ± 1.62 ^a,b^	51.55 ± 2.61 ^b^	51.72 ± 1.67 ^b^	38.01 ± 4.42 ^c^
VCL (μm/s)	61.33 ± 3.00 ^a^	50.49 ± 2.53 ^a,b^	45.43 ± 1.54 ^b^	46.42 ± 4.06 ^b,c^	32.00 ± 4.10 ^c^
VSL (μm/s)	25.90 ± 2.93 ^a^	21.74 ± 1.85 ^a^	18.01 ± 1.74 ^a,b^	20.11 ± 1.88 ^a,b^	12.49 ± 1.98 ^b^
VAP (μm/s)	35.55 ± 2.69 ^a^	29.31 ± 2.03 ^a,b^	25.23 ± 1.68 ^a,b,c^	26.94 ± 2.38 ^b,c^	17.63 ± 2.66 ^c^
BCF (Hz)	4.71 ± 0.26 ^a^	3.78 ± 0.22 ^a,b^	3.48 ± 0.25 ^a,b,c^	3.61 ± 0.31 ^b,c^	2.52 ± 0.36 ^c^
ALH (μm)	2.40 ± 0.07 ^a^	1.94 ± 0.09 ^a,b^	1.83 ± 0.07 ^b^	1.79 ± 0.15 ^b^	1.30 ± 0.17 ^c^

Sperm motility and motion kinematics are presented as mean ± SEM, *n* = 9. Values with different superscript letters (^a,b,c^) indicate significant differences between the control and treatment groups (*p* < 0.05). MOT—sperm motility (%); Rapid—rapid sperm motility (%); Medium—medium sperm motility (%); Slow—slow sperm motility (%); Progressive—progressive sperm motility (%); VCL—curvilinear velocity (μm/s); VSL—straight-line velocity (μm/s); VAP—average path velocity (μm/s); BCF—beat cross frequency (Hz); ALH—mean amplitude of head lateral displacement (μm).

## Data Availability

Not applicable.
